# Sub-microscopic malaria cases and mixed malaria infection in a remote area of high malaria endemicity in Rattanakiri province, Cambodia: implication for malaria elimination

**DOI:** 10.1186/1475-2875-9-108

**Published:** 2010-04-22

**Authors:** Nicolas Steenkeste, William O Rogers, Lucy Okell, Isabelle Jeanne, Sandra Incardona, Linda Duval, Sophy Chy, Sean Hewitt, Monidarin Chou, Duong Socheat, François-Xavier Babin, Frédéric Ariey, Christophe Rogier

**Affiliations:** 1 Unité d'Epidémiologie Moléculaire, Institut Pasteur du Cambodge, Phnom Penh, Cambodia; 2 Naval Medical Research Center Unit 2, Jakarta 10560 Indonesia; 3 London School of Hygiene & Tropical Medicine (LSHTM), London, UK; 4 Centre de recherche médicale et sanitaire (CERMES), Niamey, Niger; 5 Laboratoire de Génétique de la réponse aux infections chez l'homme, Institut Pasteur, Paris, France; 6 European Commission National Malaria Control Program, Phnom Penh, Cambodia; 7 Rodolphe Mérieux Laboratory of Cambodia, University of Health Science, Phnom Penh, Cambodia; 8 National Center for Parasitology, Entomology and Malaria Control, Phnom Penh, Cambodia; 9 Fondation Mérieux, Phnom Penh, Cambodia; 10 Equipe « Moustiques et Maladies Emergentes » - UMR 6236 - URMITE, Unité de Recherche en Biologie et Epidémiologie Parasitaires - Institut de Recherche Biomédicale des Armées, Marseille, France

## Abstract

**Background:**

Malaria microscopy and rapid diagnostic tests are insensitive for very low-density parasitaemia. This insensitivity may lead to missed asymptomatic sub-microscopic parasitaemia, a potential reservoir for infection. Similarly, mixed infections and interactions between *Plasmodium *species may be missed. The objectives were first to develop a rapid and sensitive PCR-based diagnostic method to detect low parasitaemia and mixed infections, and then to investigate the epidemiological importance of sub-microscopic and mixed infections in Rattanakiri Province, Cambodia.

**Methods:**

A new malaria diagnostic method, using restriction fragment length polymorphism analysis of the *cytochrome b *genes of the four human *Plasmodium *species and denaturing high performance liquid chromatography, has been developed. The results of this RFLP-dHPLC method have been compared to 1) traditional nested PCR amplification of the *18S rRNA *gene, 2) sequencing of the amplified fragments of the *cytochrome b *gene and 3) microscopy.

Blood spots on filter paper and Giemsa-stained blood thick smears collected in 2001 from 1,356 inhabitants of eight villages of Rattanakiri Province have been analysed by the RFLP-dHPLC method and microscopy to assess the prevalence of sub-microscopic and mixed infections.

**Results:**

The sensitivity and specificity of the new RFLP-dHPLC was similar to that of the other molecular methods. The RFLP-dHPLC method was more sensitive and specific than microscopy, particularly for detecting low-level parasitaemia and mixed infections. In Rattanakiri Province, the prevalences of *Plasmodium falciparum *and *Plasmodium vivax *were approximately two-fold and three-fold higher, respectively, by RFLP-dHPLC (59% and 15%, respectively) than by microscopy (28% and 5%, respectively). In addition, *Plasmodium ovale *and *Plasmodium malariae *were never detected by microscopy, while they were detected by RFLP-dHPLC, in 11.2% and 1.3% of the blood samples, respectively. Moreover, the proportion of mixed infections detected by RFLP-dHPLC was higher (23%) than with microscopy (8%).

**Conclusions:**

The rapid and sensitive molecular diagnosis method developed here could be considered for mass screening and ACT treatment of inhabitants of low-endemicity areas of Southeast Asia.

## Background

The development of sensitive PCR-based methods for malaria diagnosis has highlighted the low sensitivity of malaria microscopy for very low-level parasitaemia (<50 parasites/μl). The low sensitivity of microscopy may have at least two important consequences for malaria control and eradication efforts. First, asymptomatic sub-microscopic parasitaemia may serve as a reservoir for infection even when very efficient rapid diagnosis and treatment programmes have been implemented. Second, mixed infections may be overlooked when one species is present at low parasitaemia, and clinically or epidemiologically important interactions between species may be missed.

Current malaria control methods in Southeast Asia rely largely on early detection and treatment; control based on insecticide-treated bed nets (ITN) may be less effective in areas where malaria is primarily acquired not at home but by working age men engaged in mining, hunting, or logging in the forest. Since individuals with asymptomatic parasitaemia will not be identified by early detection and treatment programmes, they may continue to serve as a source of infection for vector mosquitoes, complicating control measures. It is clear that PCR-based methods can detect sub-microscopic parasitaemia [1-4]; it remains to be determined how common such cases of parasitaemia are in the field under different ecological conditions and what effect they may have on transmission.

Accurate detection of mixed infections is important for both clinical and epidemiological reasons. The four *Plasmodium *species, which commonly infect humans, have somewhat different clinical characteristics. *Plasmodium falciparum *is the most lethal species, causing severe malarial anaemia and cerebral malaria and, consequently, the vast majority of malaria deaths. *Plasmodium vivax *and *Plasmodium ovale *may persist within the liver as hypnozoites causing relapses even after treatment with blood schizonticides. *Plasmodium malariae *may cause chronic asymptomatic parasitaemia and may lead to renal failure via unclear mechanisms [5]. Finally, *Plasmodium knowlesi *is a newly emergent malaria parasite in Southeast Asia [6,7]. How these clinical characteristics are modified in mixed species infections is not clear. Voza *et al *in 2005 found a species-specific inhibition of cerebral malaria in mice coinfected with *Plasmodium spp *[8]. In human, according to several studies in Ivory Coast [9], Sri Lanka [10] and Thailand [11], the severity of infection may be modulated by mixed infections. In a study carried out in Vanuatu, *P. falciparum *appeared to be associated with a reduced *P. vivax *parasitaemia [12,13], but the reverse effect has been observed in another study [14]. There seems to be a strong interaction between these two malaria species, and *P. vivax *co-infection has even been reported to decrease treatment failures of *P. falciparum *[15]. In other studies, *P. falciparum *- *P. vivax *mixed infections affected the clinical outcome in patients [13,16]. Examples of *P. malariae *and *P. ovale *co-infections with *P. falciparum *protecting against malaria symptoms have also been reported [17]. Studies with a *Plasmodium berghei *and *Plasmodium yoelii *co-infection model in mice appear to confirm that there can be a strong cross-protective effect of mixed species infections [18]. Mixed infections may have important epidemiological effects. For example, if *P. vivax *parasitaemia is suppressed by co-infection with *P. falciparum*, then effective control of *P. falciparum *malaria in an area might be followed by an increase in *P. vivax *transmission.

Studies of mixed species infections based only on microscopy may underestimate their importance. The frequencies of minority species, such as *P. malariae *and *P. ovale*, are largely underestimated by microscopy [19-21]. PCR-based methods are more sensitive [1-4] and more readily detect mixed infections. Using PCR diagnosis, between one third and half of malaria infections in a study in Thailand were found to be mixed species infections [22].

In order to investigate the epidemiological importance of sub-microscopic and mixed species infections, a highly sensitive PCR-based diagnostic method was developed using amplification of a fragment of the *cytochrome b *gene, followed by Restriction Fragment Length Polymorphism (RFLP) analysis using Denaturing High Performance Liquid Chromatography (dHPLC) to detect amplification and restriction products. Results of the RFLP-dHPLC method were first compared to traditional PCR and microscopy. This highly sensitive method was then used to analyze samples collected in 2001 in malaria prevalence surveys in eight villages in Rattanakiri Province, a site of high malaria transmission in Cambodia, and assess the prevalence and importance of sub-microscopic and mixed infections in this population.

## Methods

### Study area and population

**Figure 1:**
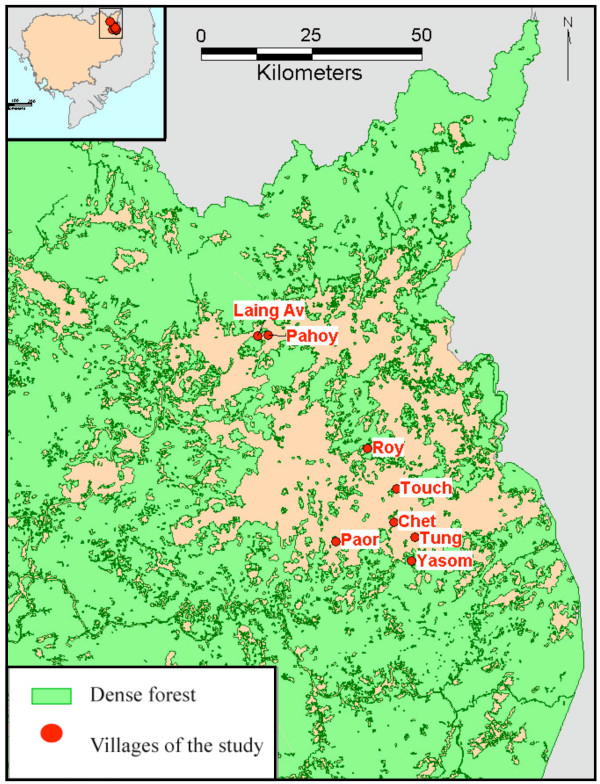
**The study area and villages in Rattanakiri province, and their location in Cambodia**. (lat/long: Yasom 13.583/107.283; Roy 13.814/107.193; Paor 13.624/107.125; Chet 13.662/107.246; Tung 13.631/107.290; Touch 13.731/107.253; Laing Av 14.046/106.964; Pahoy 14.047/106.987)

Rattanakiri province is located in North-Eastern Cambodia, close to the borders with Laos and Vietnam (Figure 1). Malaria is transmitted by *Anopheles dirus*, *Anopheles maculatus *and *Anopheles minimus *[23,24]. In 2001, the number of slide-confirmed malaria cases confirmed in Rattanakiri province by the National Malaria Control Programme (CNM) was 1,165 for *P. falciparum *(71.9%), 424 for *P. vivax *(26.1%) and 31 for *P. malariae *(2%) [25].

A cross-sectional prevalence survey was conducted in September 2001 in 36 villages classified as 'high-risk' (<1 km of the forest) of Rattanakiri province [26]. Approximately 20 μL of finger prick blood were collected on Whatman 3 M filter paper. Thin and thick blood smears were prepared, and data on age, sex and temperature were collected. Blood spots were stored at -20°C.

For the present study, eight villages from the 2001 survey were randomly selected. Those villages belonged to three ethnic minorities; respectively, Tampuon (Yasom, 204 samples; Roy, 206 samples; Chet, 204 samples and Paor, 209 samples), Jarai (Tung, 143 samples and Touch, 143 samples) and Brao (Laing Av, 132 samples and Pahoy, 134 samples).

### Microscopic diagnosis

Thin smears were fixed in methanol. Both thin and thick smears were stained with 3% Giemsa for 30 minutes at room temperature. Examination was performed by experienced microscopists at the National Centre for Parasitology, Entomology and Malaria Control in Phnom Penh. At least 100 thick film fields with 1,000× magnification were examined before a slide was considered negative. Parasite species and stages were confirmed on the thin film. Parasite densities were classified according WHO recommendations: class 1 for 1-10 parasites per 100 thick film fields; class 2 for 11-100 parasites per 100 fields; class 3 for 1-10 parasites per single field; class 4 for 11-100 parasites per single field; class 5 for > 100 parasites per single field.

### DNA extraction

Parasite DNA was extracted from a piece of 4 mm diameter of blood spots of Whatman 3 M filter paper, using the Instagene resin (Biorad, Germany) as previously described [27].

### *18S rRNA *species-specific nested PCR, standard PCR

The nested PCR method based on the *18S rRNA *gene marker [28] adapted for epidemiological studies was performed as described [29].

### Restriction Fragment Length Polymorphism (RFLP) and dHPLC

Nested PCR amplification and Alu I restriction digestion of the Plasmodium *cytochrome b *gene was performed as previously described [27,30]. Five μl of each restriction digested PCR product were injected with a 96 well auto-sampler for DHPLC analysis in the WAVE DNA Fragment Analysis System (Transgenomic, Santa Clara, CA) with a variable wave-length detector set at 260 nm. To separate DNA fragments, the analysis was performed on a DNASepTM column (Transgenomic, Inc.) at 50°C with a flow rate of 0.9 ml/min. DNA fragments were eluted with a linear gradient mixture of Buffer A: 0.1 M triethylammonium acetate (TEAA) pH 7.0 (Transgenomic) and Buffer B: 0.1 M TEAA/25% acetonitrile (ACN) pH 7.0. Elution started from 46% to 59.1% of buffer B over a period 13.5 min. The column was regenerated with the buffer D: 75% ACN pH 7.0 and re-used.

### *Cytochrome b *SNP identification

Sequencing reactions were performed on both strands of the *cytochrome b *PCR product using internal primers and ABI Prism BigDye Terminator chemistry. Sequencing reactions were run on ABI3730XL (Applied Biosystems) at Macrogen^® ^(Korea). The analysis of the sequence was performed with Seqscape software v.2.0 (Applied Biosystems). An algorithm based on 11 selected SNPs has previously been developed based on an alignment of published cytochrome b reference sequences of *P. falciparum, P. vivax, P. malariae *and *P. ovale*, allowing identification of each species [27].

### Reference genomic DNA

Genomic DNA from *P. falciparum *was extracted from a continuous culture of the 3D7 strain. DNA from the *P. vivax *Belem strain was kindly provided by Peter David (Institut Pasteur, Paris, France). *Plasmodium malariae *and *P. ovale *DNA as well as human control DNA from a non-infected person were kindly provided by Georges Snounou (Muséum d'Histoire Naturelle, Paris, France). DNA from patients with pure *P. malariae *infections were kindly provided by Eric Legrand (Institut Pasteur de Cayenne, French Guiana).

### Statistical analysis

All statistical analyses were performed using the STATA 10 software (Stata Corporation, College Station, TX). Prevalence rates and their 95% confidence intervals were estimated using the svy commands to take into account the sampling design (clustered by village). McNemar's exact test was used for paired analysis of the results of the diagnostic methods applied to the same blood samples. The associations of independent variables with malaria prevalence were tested using logistic regression models with random effects taking into account the clustered sampling design.

## Results

### RFLP-dHPLC *Plasmodium *species diagnosis validation

**Figure 2:**
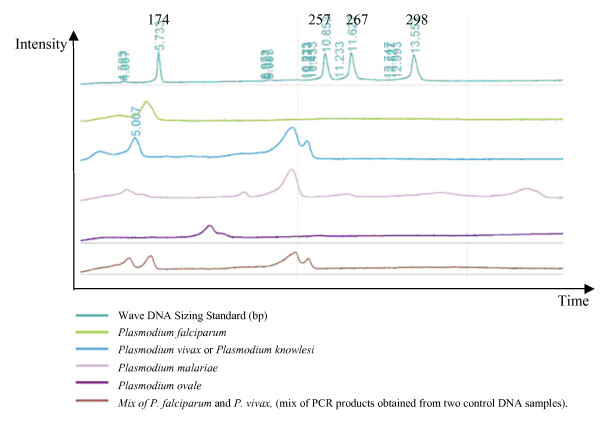
**dHPLC profile for *Plasmodium *identification**.

A new, high-throughput method for diagnosis using PCR amplification of the *cytochrome b *gene, Alu I digestion, and analysis of the resultant restriction fragment polymorphisms using dHPLC has been developed. By dispensing with gel electrophoresis, this dHPLC-based method allows more rapid, high-throughput sample processing. Figure 2 shows the dHPLC profiles produced by *Plasmodium *DNA control samples using this technique.

In order to ensure the accuracy of this approach, the RFLP-dHPLC method was first validated against the standard PCR methods (Table 1). All samples from one of the study sites (n = 134, Pahoy) were subjected to both methods. Comparison ability of the two methods to detect *Plasmodium *parasites, regardless of species, gave an agreement on 115 (21 negative and 94 positive). Eight samples were positive according to RFLP-dHPLC, but not according to the standard PCR and 11 were positive by the standard PCR, but not by RFLP-dHPLC. There was no evidence for the superiority of either method in detecting *Plasmodium *parasites (McNemar's exact test p = 0.65). The only difference in species identification between the two methods occurred in cases in which one test detected a mixed species infection and the other test identified only the predominant species. For example, the standard PCR approach identified eight mixed infections among 56 infections identified as *P. falciparum *mono-infections by dHPLC, while dHPLC identified four mixed infections among 48 infections identified as *P. falciparum *mono-infections by standard PCR. Twenty-three mixed infections were detected by both techniques. There was no evidence for superiority of either technique in detecting mixed species infections including *P. falciparum *(McNemar's exact test p = 0.39).

**Table 1:** Comparison of results by RFLP-dHPLC and by standard PCR method in Pahoy

**RFLP-dHPLC**	**Standard PCR**											
	
	**neg**	**F**	**V**	**O**	**FM**	**FO**	**FV**	**VM**	**FVM**	**FVO**	**FVMO**	**Total**
neg	21	4	4	0	0	0	0	0	0	0	0	29
												
F	8	40	0	0	0	0	6	0	1	1	0	56
V	1	0	6	0	0	0	2	1	0	0	0	10
M	2	0	0	0	0	0	0	2	0	0	0	4
O	0	0	0	0	0	1	0	0	0	0	0	1
												
FM	0	1	0	0	4	0	0	0	2	0	0	7
FO	0	0	0	0	0	1	0	0	0	0	0	1
FV	0	3	2	0	0	0	10	0	0	0	2	17
VM	0	0	0	0	0	0	0	0	1	0	0	1
VO	0	0	0	2	0	0	0	0	0	0	0	2
												
FVM	0	0	0	0	0	0	0	0	3	0	1	4
FVO	0	0	0	0	0	0	0	0	0	2	0	2
												
Total	32	48	12	2	4	2	18	3	7	3	3	134

Because of the same pattern found for *P. vivax *and *P. knowlesi *in dHPLC technique, all isolates found to be infected by *P. vivax *were sequenced. No *P. knowlesi *infection was found.

Since the RFLP-dHPLC method did not appear inferior to standard PCR diagnosis, validation was done on the sequence specificity of the new method by comparing the species identification made by RFLP-dHPLC detection with the results of *cytochrome b *SNP analysis as determined by direct DNA sequencing of *cytochrome b *PCR products [27]. For mono-infections there were no discrepancies between the methods. In the case of mixed infections, each technique occasionally missed a minor species identified by the other (Table 2). Overall, the two techniques gave the same species identification, including mixed species, in 85/99 (86%) of cases. In seven cases RFLP-dHPLC identified mixed infections missed by *cytochrome b *SNP analysis; in seven cases, *cytochrome b *SNP analysis identified mixed infections missed by RFLP-dHPLC. Since the RFLP-dHPLC method appeared no less sensitive than standard PCR and identified mixed infections as effectively as *cytochrome b *SNP analysis, the RFLP-dHPLC method was used to assess the prevalence of sub-microscopic and mixed infections in samples from eight villages in Rattanakiri.

**Table 2:** Comparison of results by RFLP-dHPLC and by sequence analysis of SNP from *cytochrome b *gene in Pahoy

**RFLP-dHPLC**	**sequence analysis of SNP from *cytochrome b *gene**											
	
	**neg**	**F**	**V**	**M**	**FM**	**FO**	**FV**	**VM**	**VO**	**FVM**	**FVO**	**Total**
neg	21	3	2	0	0	0	3	0	0	0	0	29
												
F	5	50	0	0	1	0	0	0	0	0	0	56
V	0	0	8	0	0	0	2	0	0	0	0	10
M	0	0	0	3	0	0	0	1	0	0	0	4
O	0	0	0	0	0	1	0	0	0	0	0	1
												
FM	0	1	0	1	4	0	0	0	0	1	0	7
FO	1	0	0	0	0	0	0	0	0	0	0	1
FV	0	2	2	0	0	0	12	0	0	1	0	17
VM	0	0	0	0	0	0	0	1	0	0	0	1
VO	0	0	1	0	0	0	0	0	1	0	0	2
												
FVM	0	0	0	0	0	0	0	0	0	4	0	4
FVO	0	0	0	0	0	0	0	0	0	0	2	2
												
Total	27	56	13	4	5	1	17	2	1	6	2	134

### Comparison of RFLP-dHPLC and microscopy results

A comparison between the numbers of *Plasmodium *infections detected by microscopy and by the RFLP-dHPLC method was done on 1,356 samples collected from eight villages in Rattanakiri province (Table 3). The prevalence of *Plasmodium *infections measured by the RFLP-dHPLC method (68.4%, 95%CI: 62.7% - 74.1%) was more than twice that measured by microscopy (30.7%, 95%CI: 24.9% - 36.4%).

**Table 3:** Comparison between microscopy and RFLP-dHPLC diagnostic methods

**RFLP-dHPLC**	**Microscopy**				
	
	**neg**	**F**	**V**	**FV**	**Total**
neg	388	32	5	3	428
					
F	331	241	6	13	591
V	50	5	8	4	67
M	35	2	1	1	39
O	3	1	0	0	4
					
FM	59	24	3	2	88
FO	2	3	0	1	6
FV	49	35	10	7	101
VM	5	2	0	1	8
VO	4	0	0	0	4
					
FVM	11	4	1	0	16
FVO	2	1	0	0	3
					
FVMO	1	0	0	0	1
					
Total	940	350	34	32	1356

Regardless of species, the two techniques agreed on 764 samples (376 positive and 388 negative according to both techniques). Nevertheless, 552 specimens negative by microscopy were positive by RFLP-dHPLC, and 40 negative by RFLP-dHPLC were positive by microscopy: 32 *P. falciparum*, five *P. vivax *and three mixed infections *P. falciparum *with *P. vivax*; the *P. falciparum *density is distributed as follow: 16 in class 1 of parasite densities, 14 in class 2, four in class 3 and one in class 4. The RFLP-dHPLC method identified many more infections than microscopy (McNemar's exact test p < 0.0001).

Table 4 and 5 show the prevalence of malaria infection in the individual villages as determined by both microscopy and RFLP-dHPLC. The prevalence of *P. falciparum *and *P. vivax *was approximately two-fold and three-fold higher, respectively, when measured by the RFLP-dHPLC (*P. falciparum*: 59.4%, 95%CI: 52.2% - 66.6%; *P. vivax*: 14.7%, 95%CI: 10.8% - 18.7%) than by microscopy (*P. falciparum*: 28.2%, 95%CI: 21.6% - 34.7%; *P. vivax*: 4.8%, 95%CI: 3.1% - 6.6%). In addition, *P. ovale *and *P. malariae *were never detected by microscopy while they were detected by RFLP-dHPLC (*P. malariae*: 11.2%, 95%CI: 8.2% - 14.2%; *P. ovale*: 1.3%, 95%CI: 0.04% - 2.6%).

**Table 4:** Prevalence of *Plasmodium spp*. by village diagnosed by microscopy (*P. spp*.: All *Plasmodium *species; *Pf*: *Plasmodium falciparum*; *Pv*: *Plasmodium vivax*; *Pm*: *Plasmodium malariae*; *Po*: *Plasmodium ovale*)

	**Microscopy**						
		
		***P. spp***		***Pf***		***Pv***	
				
**Village**	**N**	**%**	**IC95%**	**%**	**IC95%**	**%**	**IC95%**
							
Yasom	204	38,7%	(32,0-45,8)	36,8%	(30,1-43,8)	8,3%	(4,9-13,0)
Roy	205	37,1%	(30,4-44,1)	33,2%	(26,8-40,1)	7,8%	(4,5-12,4)
Paor	209	25,4%	(19,6-31,8)	23,0%	(17,4-29,3)	3,8%	(1,7-7,4)
Chet	202	27,7%	(21,7-34,4)	26,2%	(20,3-32,9)	3,0%	(1,1-6,4)
							
Tung	141	20,6%	(14,2-28,2)	14,2%	(8,9-21,1)	9,2%	(5,0-15,3)
Touch	143	35,0%	(27,2-43,4)	34,3%	(26,5-42,7)	1,4%	(0,2-5,0)
							
Laing Av	118	28,8%	(20,8-37,9)	26,3%	(18,6-35,2)	2,5%	(0,5-7,3)
Pahoy	134	29,1%	(21,6-37,6)	28,4%	(20,9-36,8)	0,7%	(0,0-4,1)

**Table 5:** Prevalence of *Plasmodium spp*. by village diagnosed by RFLP-dHPLC (*P. spp*.: All *Plasmodium *species; *Pf*: *Plasmodium falciparum*; *Pv*: *Plasmodium vivax*; *Pm*: *Plasmodium malariae*; *Po*: *Plasmodium ovale*)

	**RFLP-dHPLC**									
	
	***P. spp***.		***Pf***		***Pv***		***Pm***		***Po***	
					
**Village**	**%**	**IC95%**	**%**	**IC95%**	**%**	**IC95%**	**%**	**IC95%**	**%**	**IC95%**
										
Yasom	71,6%	(64,8-77,6)	66,7%	(59,7-73,1)	14,2%	(9,7-19,8)	10,8%	(6,9-15,9)	2,0%	(0,5-4,9)
Roy	65,4%	(58,4-71,9)	56,1%	(49,0-63,0)	11,2%	(7,2-16,4)	9,8%	(6,1-14,7)	2,0%	(0,5-4,9)
Paor	68,9%	(62,1-75,1)	60,3%	(53,3-67,0)	12,4%	(8,3-17,7)	15,3%	(10,7-20,9)	0,0%	(0,0-1,7)
Chet	66,3%	(59,4-72,8)	58,9%	(51,8-65,8)	11,9%	(7,8-17,2)	5,9%	(3,1-10,1)	0,0%	(0,0-1,8)
										
Tung	53,2%	(44,6-61,6)	39,0%	(30,9-47,6)	16,3%	(10,6-23,5)	12,1%	(7,2-18,6)	1,4%	(0,2-5,0)
Touch	69,9%	(61,7-77,3)	61,5%	(53,0-69,5)	16,8%	(11,1-23,9)	13,3%	(8,2-20,0)	1,4%	(0,2-5,0)
										
Laing Av	76,3%	(67,6-83,6)	67,8%	(58,6-76,1)	12,7%	(7,3-20,1)	11,9%	(6,6-19,1)	0,0%	(0,0-3,1)
Pahoy	78,4%	(70,4-85,0)	64,9%	(56,2-73,0)	26,9%	(19,6-35,2)	11,9%	(7,0-18,7)	4,5%	(1,7-9,5)

### Prevalence of sub-microscopic *P. falciparum *infection

**Figure 3:**
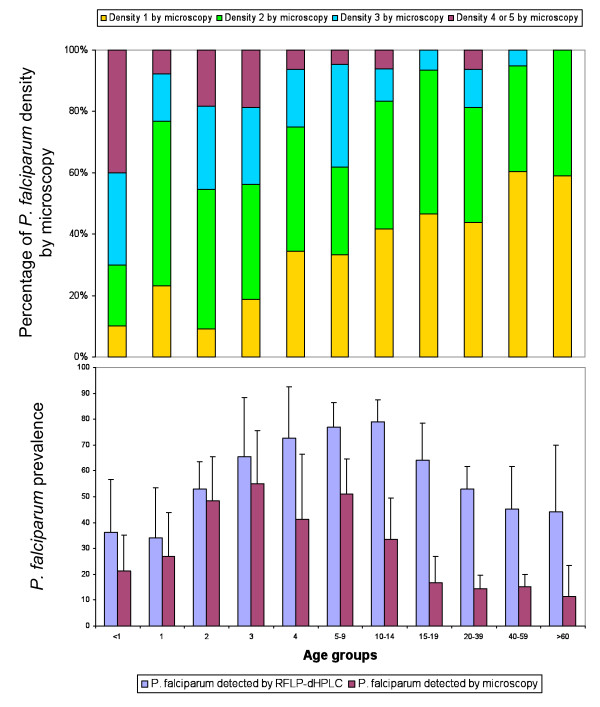
**Prevalence of *P. falciparum *by age diagnosed by microscopy and RFLP-dHPLC**. Density class 1 for 1--10 parasites per 100 thick film fields; Density class 2 for 11--100 parasites per 100 fields; Density class 3 for 1--10 parasites per single field; Density class 4 for 11--100 parasites per single field; Density class 5 for > 100 parasites per single field (WHO, Basic Malaria Microscopy, Geneva, Switzerland)

In subjects less than five years old, microscopy and RFLP-dHPLC gave similar *P. falciparum *prevalences. In subjects aged five years or older, the *P. falciparum *prevalence was higher when estimated by RFLP-dHPLC than microscopy (Figure 3). The *P. falciparum *parasite densities estimated by microscopy decrease significantly with increasing age. No such association of age with prevalence or parasite density was found for the three other *Plasmodium *species.

### Characterization of mixed infection

Due to the higher sensitivity of RFLP-dHPLC, the proportion of mixed infections was significantly higher with this method (24.5%, 227/928) than with microscopy (7.7%, 32/416) (Table 3). The frequency of mixed infections detected by RFLP-dHPLC did not vary significantly depending on the positivity of the microscopy (p = 0.75) or the parasite density (p = 0.91) estimated by microscopy.

### Sex and ethnic groups

*Plasmodium falciparum *was more prevalent in males than in females (p < 0.001). No such association was found for the other malaria species (Table 6). Between ethnic groups, the prevalence of *P. falciparum *and *P. vivax*, but not of *P. malariae *or *P. ovale*, differed significantly (Table 6). The prevalence of *P. falciparum *was significantly higher in the Brao than in the Tampoun and in the Tampoun than in the Jaray ethnic groups, and the prevalence of *P. vivax *was significantly higher in the Brao than in the Tampoun, without significant differences between the Jaray and the other ethnic groups.

**Table 6:** Associations of *P. falciparum *and *P. vivax *infection with other Plasmodium infections and demographics; results of logistic regression analysis allowing for random village effects. N = 1319 individuals from 8 villages

		*P. falciparum infections*						*P. vivax infections*					
			Prevalence						Prevalence				
	N	positive	rate	OR	IC95%		p-value	positive	rate	OR	IC95%		p-value
*P. falciparum infection*													
Not detected*	540							77	14,3%	1,00			
Detected*	779							114	14,6%	0,92	0,66	1,30	0,647
*P. vivax infection*													
Not detected*	1128	665	59,0%	1,00									
Detected*	191	114	59,7%	0,90	0,64	1,26	0,535						
*P. malariae infection*													
Not detected*	1173	680	58,0%	1,00				168	14,3%	1,00			
Detected*	146	99	67,8%	1,34	0,91	1,99	0,138	23	15,8%	1,17	0,71	1,90	0,540
*P. ovale infection*													
Not detected*	1301	769	59,1%	1,00				183	14,1%	1,00			
Detected*	18	10	55,6%	0,63	0,23	1,73	0,367	8	44,4%	4,35	1,64	11,49	0,003
Sex													
Female	723	400	55,3%	1,00				92	12,7%	1,00			
Male	596	379	63,6%	1,54	1,21	1,96	<0,001	99	16,6%	1,35	0,98	1,86	0,064
Age groups (years)													
< 1	50	14	28,0%	1,00				7	14,0%	1,00			
1	41	14	34,1%	1,69	0,67	4,21	0,264	14	34,1%	3,77	1,33	10,71	0,013
2	66	35	53,0%	3,35	1,51	7,46	0,003	8	12,1%	0,89	0,30	2,68	0,838
3	58	38	65,5%	6,06	2,61	14,08	<0,001	12	20,7%	1,71	0,60	4,85	0,313
4	51	37	72,5%	7,99	3,29	19,43	<0,001	7	13,7%	1,02	0,32	3,20	0,978
5-9	225	173	76,9%	10,85	5,33	22,10	<0,001	41	18,2%	1,45	0,59	3,57	0,418
10-14	134	106	79,1%	12,78	5,93	27,55	<0,001	27	20,1%	1,66	0,65	4,27	0,290
15-19	95	61	64,2%	6,21	2,87	13,43	<0,001	9	9,5%	0,69	0,23	2,02	0,494
20-39	401	212	52,9%	3,41	1,75	6,62	<0,001	46	11,5%	0,86	0,36	2,07	0,742
40-60	146	66	45,2%	2,43	1,19	4,96	0,014	15	10,3%	0,72	0,27	1,91	0,513
>60	52	23	44,2%	2,48	1,06	5,79	0,035	5	9,6%	0,64	0,19	2,23	0,488
Ethnic groups													
Jaray	269	133	49,4%	1,00				44	16,4%	1,38	0,92	2,06	0,115
Tampoun	801	482	60,2%	1,86	1,19	2,90	0,007	96	12,0%	1,00			
Brao	249	164	65,9%	2,60	1,52	4,47	0,001	51	20,5%	2,03	1,38	2,99	<0,001

* by RFLP-dHPLC

### Evidence of non-random species associations

Controlling for age and ethnic group in multivariate random effects logistic regression models, *P. vivax *infection was significantly associated with *P. ovale *infection (OR = 4.35, IC95 = 1.64 - 11.49, p = 0.003) (Table 6). There was also an association between *P. falciparum *and *P. malariae *infections (unadjusted OR = 1.61, IC95 = 1.12 - 2.32, p = 0.010) that was no more significant when controlling for age (adjusted OR = 1.34, IC95 = 0.91 - 1.99, p = 0.138). There was no association between *P. falciparum *and *P. vivax *infection (p = 0.535).

## Discussion

The detection of *Plasmodium *species at very low parasitaemia is difficult and requires a molecular approach. The performance of a relatively simple and semi-automated technique has been evaluated here. It is a highly sensitive PCR-based diagnostic method using amplification of a fragment of the *cytochrome b *gene, followed by RFLP analysis using dHPLC to detect amplification and restriction products. Compared to the well established PCR typing of the *18S rRNA *gene for species detection, this method appeared reliable, sensitive (multi copy number of the *cytochrome b *gene), specific and rapid (two PCR instead of five) and give the ability to amplified large number of species [27]. The dHPLC semi-automated analysis allowed the screening of a 96-well plate in 48 h and to diagnose 1,356 samples in less than one month.

Use of the RFLP-dHPLC method revealed a high prevalence of sub-microscopic infections. The overall prevalence detected by the RFLP-dHPLC method was twice that detected by microscopy (68.4% versus 30.7%). Interestingly, the proportion of sub-microscopic *P. falciparum *infections, was higher in older age groups. It is possible that acquired immunity favours maintenance of sub-microscopic, asymptomatic infections. This substantial population of adults with sub-microscopic, asymptomatic *P. falciparum *infections may represent a significant challenge to malaria control programmes. Such individuals will not seek treatment and even if they were included in a mass screening and treatment campaign, their parasitaemia would remain invisible to light microscopy or rapid diagnostic tests. Moreover, sub-microscopic *P. falciparum *gametocyte densities may contribute importantly to mosquito infections and thus to maintaining transmission [27]. In order to have a major impact on malaria transmission, mass screening and treatment campaigns may need to use molecular screening techniques capable of identifying sub-microscopic parasitaemia.

The RFLP-dHPLC method detected many more mixed species infections than did microscopy (Table 3). Indeed, 24.5% of all malaria infections in the Rattanakiri survey included more than one species. No association was found between the level of parasitaemia and the chance that the infection would be mixed. Co-infection with *P. vivax *and *P. ovale *was more common than would be expected based on the individual prevalences of these species (OR = 4.35, IC95 = 1.64 - 11.49, p = 0.003). This association could be attributed to cross-immunity that determines susceptibility to both infections [31] or to the exposure to infective bites of common vectors that transmit both species. An alternative explanation might be that both these species can produce relapses from hypnozoites. Similarly, *P. falciparum *and *P. malariae *infection were associated (OR = 1.61, IC95 = 1.12 - 2.32, p = 0.010). There was, however no association between *P. vivax *and *P. falciparum *infection.

*Plasmodium falciparum *and *P. vivax *infections were found at different prevalences in the different ethnic groups. Since the different groups lived in different individual villages, it is not possible to decide whether the observed differences reflect different genetic susceptibilities, as observed for some African ethnic groups [32] or simply to different local transmission intensities or other local factors.

## Conclusions

Rapid diagnosis and treatment of malaria cases, vector control and protection against mosquito bites are the cornerstone of the malaria control strategy in Cambodia. Sub-microscopic infections may provide a reservoir of infection, which maintains transmission. This phenomenon has already been highlighted in Guinea Bissau [1] in Brazil [33] and Gabon [34]. The high prevalence of sub-microscopic parasitaemia within Rattanakiri Province, a site of higher malaria transmission in Cambodia, is here shown. Such infections may hamper elimination efforts, since they are asymptomatic and would not be efficiently detected by conventional diagnosis methods, *i.e*. microscopy or rapid diagnostic tests. Thus, the rapid and sensitive molecular diagnosis method developed here could be considered for mass screening and ACT treatment of inhabitants of low-endemicity areas of Southeast Asia to face the urgent need to eradicate malaria in Cambodia due to emerging artemisinin resistance in the country.

## Competing interests

The authors declare that they have no competing interests.

## Authors' contributions

NS, LD and FA conceived and designed the genus-specific nested PCR and SNP identification based on the *cytochrome b *gene, SH and SD lead the field work, NS, SI, MC, FX, SC managed the experimental procedure and performed the laboratory work, NS, CR, WR, LO, FA participated in the statistical analyses, NS, IJ participated in SIG work. NS, WR, LO, IJ, SI, LD, SC, SH, DS, FB, FA, CR drafted and critically revised the manuscript. All authors read and approved manuscript.

## Ethical approval

Ethical approval for this study was granted by the National Ethics Committee of the Kingdom of Cambodia.
